# Evaluation of Coronary Arteries in Non-Ischemic Cardiomyopathies: A Case Report

**Published:** 2016-10-03

**Authors:** Farveh Vakilian, Mahmood Mohamadzadeh Shabestari, Ahmad Amin, Soheila Chamanian, Toktam Moghiman

**Affiliations:** 1 *Atherosclerosis Prevention Research Center, Imam Reza Hospital, Mashhad University of Medical Sciences, Mashhad, Iran.*; 2 *Rajaie Cardiovascular, Medical and Research Center, Iran University of Medical Sciences, Tehran, Iran.*

**Keywords:** *Coronary vessels*, *Cardiomyopathies*, *Heart ventricles*, *Diagnosis*

## Abstract

Arrhythmogenic right ventricular dysplasia/cardiomyopathy (ARVD/C) is a congenital cardiac disease with myocardial involvement, most probably right ventricular (RV) dysfunction, accounting for 20% of sudden cardiac deaths. Characterized by the fibro-fatty infiltration of the RV free wall, ARVD/C presents in adolescents with ventricular arrhythmias and heart failure symptoms and as biventricular failure in adults. The coronary risk in these patients is not clear.

We present an incidental finding: the left anterior descending artery cut-off in a middle-aged man with ARVD/C. He had been under treatment for heart failure symptoms, which had decompensated frequently commencing 6 months earlier, and therefore he was scheduled for stem cell injection. He had no chest pain or coronary artery disease risk factors. Two-dimensional transthoracic echocardiography demonstrated RV enlargement with moderate to severe dysfunction and left ventricular ejection fraction (LVEF) of 35-40%, which was 45-50% two years before. Selective coronary angiography performed 8 years previously was normal but a new one revealed the cut-off of the left anterior descending artery at the proximal portion, for which percutaneous coronary intervention was performed and showed no significant lesion in the other vessels.

One should consider coronary artery disease in uncontrolled heart failure with LVEF reduction, even in the absence of typical chest pain. It may not be the natural course of the underlying disease.

## Introduction

Arrhythmogenic right ventricular dysplasia/cardiomyopathy (ARVD/C) is a type of genetic cardiomyopathy first described in 1977 by Fontaine. It is characterized by the fibro-fatty infiltration of the right ventricle, which accounts for 20% of sudden cardiac deaths.^1^ Due to certain abnormalities in heart-muscle proteins, they cannot keep the heart muscles together in stress-like exercises. The cells become detached and die and are replaced with fibrous fatty tissue. As a result of these changes to the structure of the heart muscle, the walls of the ventricle become thin and stretched, which means that the heart cannot pump effectively.^1^^, ^^2^ Also, because of the changes to the heart muscle cells, the normal passage of electrical impulses through the heart is interrupted or altered, which can cause life-threatening arrhythmias (abnormal heart rhythms) and sudden cardiac death. It manifests in teenage years with arrhythmias, dyspnea, and edema.

There are different options to establish a definite diagnosis but recently, echocardiography and especially cardiovascular magnetic resonance have been the most helpful. Patients should receive an implantable cardioverter-defibrillator (ICD) and antiarrhythmic drugs and, in addition, conventional heart failure treatment should be considered.^3^^, ^^4^

## Case Report

A 49-year-old Asian male was referred to the heart failure clinic due to exertional dyspnea, edema, and fatigue of 2 years' duration, which had exacerbated recently. He was a known case of ARVD/C. Eleven years before in 2001, he was admitted with sustained ventricular tachycardia; and after treatment with antiarrhythmic drugs, he was referred to Rajaei Cardiovascular, Medical and Research Center. The diagnosis was ARVD/C according to his echocardiography data: left ventricular ejection fraction of 55%, moderate right ventricular enlargement, mild right ventricular dysfunction, normal pulmonary artery pressure, mild mitral regurgitation, and moderate tricuspid regurgitation. Coronary angiography was normal and cardiovascular magnetic resonance was not available at the time. An ICD was inserted and antiarrhythmic drugs were tapered within 2 months.

He had been well since then with no events until he visited our clinic with complaints of dyspnea (New York Heart Association [NYHA] class II) and edema, which are signs of right ventricular failure. He had no chest pain, no coronary artery disease risk factors, and no history smoking. The jugular venous pressure was elevated in the upper neck, no specific sounds were heard in heart examination, and the lungs were clear. Electrocardiography revealed a normal sinus rhythm (heart rate =45), poor R wave progression, no ST-T changes, and a narrow QRS (90 msec). Echocardiography was done once again and showed left ventricular ejection fraction of 40-45%, moderate right ventricular enlargement, moderate right ventricular dysfunction, no significant regional wall motion abnormalities, mild mitral regurgitation, moderate tricuspid regurgitation, and systolic pulmonary artery pressure of 35-40 mm Hg. His laboratory data were as follows: normal fasting blood sugar of 95 mg/dL; pro-blood natriuretic peptide (proBNP) of 2357 pg/mL; total cholesterol of 195 mg/dL; low-density cholesterol of 125 mg/dL; high-density cholesterol of 35 mg/dL; triglyceride of 157 mg/dL; normal cell blood count; urea of 45 mg/dL; creatinine of 1.2 mg/dL; sodium of 136 mg/dL; potassium of 4.5 mg/dL; aspartate amino transferase of 35 mg/dL; alanine amino tranferase of 48 mg/dL; total bilirubin of 2.4 mg/dL; direct bilirubin of 0.9 mg/dL; uric acid of 8.9 mg/dL; thyroid stimulating hormone of 5.7 mg/dL ; and ferritin of 213 mg/dL.

In terms of the natural history of ARVD/C, the patient was in phase 3. Therefore, medication with Lisinopril, Carvedilol, Furosemide, Spironolactone, Folic Acid, and Sildenafil was commenced. Additionally, the ICD was programmed on a heart rate of 65. After 2 weeks, Lisinopril was discontinued because of hypotension, so digoxin was started after reassurance of the heart rate.

He was in good physical condition (NYHA class I-II) on the above-mentioned treatment for 9 months, during which he was followed up every 2 months. Afterward, he referred to our clinic with edema and dyspnea. Furosemide was changed to Torasemide; his condition improved but he experienced symptoms relapse (NYHA class III) with no chest pain. Echocardiography and laboratory tests were repeated and the ICD was reprogrammed. There were no complications: proBNP increased to 2970, urea and liver enzymes rose mildly, and there was no anemia.

The patient had left ventricular ejection fraction of 35-40%, mild hypokinesia in the septum, no change in the right ventricular size, and no pericardial effusion. Once more, based on the natural course of the disease, he was graded as phase 4. Accordingly, decision was made to evaluate him for heart transplantation. However, he was first candidated for stem cell injection in the hope of repairing the damaged myocardium. After he underwent bone marrow aspiration and mononuclear cell harvest in ROYAN Institute, he was transferred to our hospital, where angiography unexpectedly showed that the left anterior descending artery was cut off, while the other vessels were normal. Because it was not possible to perform cardiovascular magnetic resonance, the evaluation of the right ventricular function and left ventricular viability was done via stress echocardiography. The right ventricular data were similar to those mentioned before, and there was significant viability in the left anterior descending artery territory, so angioplasty was done on the left anterior descending artery.

On the last follow-up, the patient was in good condition (NYHA class I) and his jugular venous pressure and edema had improved. He is still on heart failure treatment, and the progression of right ventricular failure symptoms is not quite predictable.

## Discussion

ARVD/C is an uncommon condition in which the myocardium is the site mainly involved. This condition has four phases: 1) a concealed phase in which the patient is asymptomatic; 2) overt electrical instability; 3) signs and symptoms of right ventricular failure; and 4) frank biventricular failure, which occurs in a minority of patients protected from sudden cardiac death.^2^^, ^^5^ Coronary disease is rarely reported.^6^ Many cardiomyopathy patients reach the coronary artery disease age with some coronary risk factors, yet the possibility of atherosclerosis development cannot be predicted. The main chief complaint in heart failure is dyspnea; some patients have chest pain which can be due to pericardial involvement or low cardiac output, whereas others have no chest pain.

According to the guidelines, coronary angiography is indicated when there is typical chest pain or more than two coronary risk factors.^7^ However, is it preferable to consider coronary angiography when the patient has decompensated heart failure or has been admitted repeatedly in spite of full medical treatment. Moreover, it is advisable to take into account coronary disease when there is a reduction in the left ventricular ejection fraction, although biventricular failure is in the natural course of the disease.

## Conclusion

Arrhythmogenic right ventricular dysplasia/cardiomyopathy, which is now known as arrhythmogenic cardiomyopathy, is a progressively debilitating disease. As the patients are likely to reach the coronary disease age, it is prudent that coronary risk factors and coronary artery disease be considered in these patients. Future studies are required to determine whether revascularization can be beneficial or whether efforts should be solely focused on heart failure.

## Notes:

***This paper should be cited as:*** Vakilian F, Mohamadzadeh Shabestari M, Amin A, Chamanian S, Moghiman T. Evaluation of Coronary Arteries in Non-Ischemic Cardiomyopathies: A Case Report. J Teh Univ Heart Ctr 2016;11(4):192-194.
